# Practitioners' perspectives on spatial reasoning in educational practice from birth to 7 years

**DOI:** 10.1111/bjep.12579

**Published:** 2023-02-20

**Authors:** Kathryn E. Bates, Ashley Y. Williams, Katie A. Gilligan‐Lee, Catherine Gripton, Andrea Lancaster, Helen Williams, Alison Borthwick, Sue Gifford, Emily K. Farran

**Affiliations:** ^1^ Department of Psychology, Institute of Psychology, Psychiatry and Neuroscience Kings College London London UK; ^2^ School of Psychology University of Surrey Guildford UK; ^3^ Centre for Educational Neuroscience University of London London UK; ^4^ School of Psychology Univesity College Dublin Dublin Ireland; ^5^ School of Education University of Nottingham Nottingham UK; ^6^ EY Mathematics Specialist Consultant Cornwall UK; ^7^ International Mathematics and Education Advisor Norwich UK; ^8^ School of Education University of Roehampton Roehampton UK

**Keywords:** early childhood, mathematics, practitioners, spatial reasoning, spatial thinking, translating research

## Abstract

**Background:**

There is a growing evidence base for the importance of spatial reasoning for the development of mathematics. However, the extent to which this translates into practice is unknown.

**Aims:**

We aimed to understand practitioners' perspectives on their understanding of spatial reasoning, the extent to which they recognize and implement spatial activities in their practice, and the barriers and opportunities to support spatial reasoning in the practice setting.

**Sample:**

Study 1 (questionnaire) included 94 participants and Study 2 (focus groups) consisted of nine participants. Participants were educational practitioners working with children from birth to 7 years.

**Methods:**

The study was mixed methods and included a questionnaire (Study 1) and a series of focus groups (Study 2).

**Results:**

We found that whilst practitioners engage in a variety of activities that support spatial reasoning, most practitioners reported little confidence in their understanding of what spatial reasoning is.

**Conclusion:**

Informative and accessible resources are needed to broaden understanding of the definition of spatial reasoning and to outline opportunities to support spatial reasoning.

## INTRODUCTION

Developing proficient spatial skills is an important contributor to mathematics achievement throughout early childhood (Gilligan, Hodgkiss, et al., 2019; Gilligan, Thomas, & Farran, 2020; Hawes & Ansari, 2020; Verdine et al., 2014). However, from an educational policy perspective, the development of spatial skills is often overlooked in education curricula, including mathematics curricula (Gilligan‐Lee et al., 2022). Furthermore, it has been indicated that practitioners receive little instruction during pre‐service training on the importance of spatial reasoning and how best to embed spatial activities into their practice (Davis & Spatial Reasoning Study Group, 2015). Consequently, practitioners' use of spatial reasoning in the practice setting (e.g., childminder's home, nursery, school) is largely undocumented, and it is unclear the extent to which practitioners are aware of the specific spatial and mathematics benefits of many of the activities that they might instinctively use in their practice. Due to the established connection between practitioner beliefs and their practice (Schoen & LaVenia, 2019), knowledge of practitioner awareness of the relationships between spatial skills and mathematics is essential. There is a need for researchers to work alongside practitioners to translate research into practice, and the first step to developing effective and accessible resources for practitioners is to better understand practitioner's perspectives. Using a participatory approach, this mixed methods paper outlines practitioners' understanding of spatial reasoning, the extent to which they recognize and implement spatial activities in their practice, as well as the opportunities and barriers to implementing spatial reasoning.

### What is spatial reasoning?

Note that throughout the paper, we use the term ‘spatial reasoning’ rather than the term ‘spatial thinking’. Although spatial thinking is more common in the research literature, spatial reasoning is the term currently used in the statutory educational programme for mathematics from birth to five years in England (Department for Education, 2021). Therefore, in this paper spatial reasoning is used in a synonymous way to the meaning of spatial thinking.

We refer to spatial reasoning as the ability to mentally manipulate objects and to understand the relations between objects and oneself (Gifford et al., 2022). Spatial reasoning can be broadly sub‐divided into two subdomains, intrinsic and extrinsic skills (Newcombe & Shipley, 2015; Uttal et al., 2013). Intrinsic skills are within‐object and, therefore, involve the manipulation of objects and their parts, that is mentally representing and transforming objects to interpret their size and orientation. Extrinsic skills are between‐object and thus encompass the ability to navigate and to understand the spatial relations between objects (Newcombe, 2018; Newcombe & Shipley, 2015; Uttal et al., 2013). This classification of intrinsic versus extrinsic spatial skill has been supported at the neural level (Wraga et al., 2005) and throughout child development (Hodgkiss et al., 2021; Mix et al., 2018). More recently, Newcombe (2018) outlined evolutionary evidence to distinguish intrinsic spatial skills (that evolved for tool use) from extrinsic spatial skills (that evolved for navigation). Newcombe (2018) further highlighted a third aspect of spatial thinking, spatialization, which encompasses the symbol systems required for thinking and reasoning about space, for example spatial language, gesture, diagrams and maps. These spatial tools help to support spatial reasoning across both intrinsic and extrinsic domains. The current study is designed to encompass these three types of spatial reasoning: intrinsic spatial skills, extrinsic spatial skills and the use of spatial language and gesture as representative symbol systems. These types of spatial skills are exemplified throughout development, starting from birth as infants begin to manipulate objects (intrinsic) and explore the world around them (extrinsic), and begin to communicate spatially, such as lifting their arms up and using the word ‘up’, to be picked up (spatial symbols) (see Oudgenoeg‐Paz et al., 2015).

### Associations between spatial reasoning and mathematics

There is strong longitudinal and cross‐sectional evidence that spatial and mathematics abilities are associated in childhood (Atit et al., 2021; Gilligan et al., 2017). For example, skills such as mental rotation and block construction performance have been shown to be associated with later mathematics competence (Gilligan et al., 2017; Gunderson et al., 2012; Verdine et al., 2017). Additionally, there is evidence that spatial ability is particularly malleable in childhood (Hawes et al., 2022; Uttal et al., 2013) and that the association between spatial skill and mathematics is causal, with numerous studies finding that spatial ability training also leads to improvements in mathematics performance (Hawes et al., 2022). This evidence demonstrates not only that spatial reasoning is associated with mathematics, but also suggests that it is an important foundation for the development of number and mathematics skills. Below, we discuss this association for each of the three types of spatial ability, intrinsic and extrinsic spatial skills and spatial symbols (specifically spatial language and gesture) in turn.

#### Intrinsic spatial skills

A 2022 meta‐analysis found that spatial training using concrete materials (physical objects) led to larger improvements in mathematics compared to training that did not use a concrete component (Hawes et al., 2022). These concrete materials included objects such as tiles, blocks, multi‐link cubes and magnetic shapes. For example, in one classroom‐based intervention with 5‐ to 7‐year‐olds, training, which targeted intrinsic skills using materials such as multi‐link cubes and magnetic shapes, was found to be effective at improving both spatial and mathematics ability (Hawes et al., 2017). Block building and puzzle training in pre‐school children has also been shown to be effective (Schmitt et al., 2018) and appears to be particularly beneficial for pre‐school children from disadvantaged backgrounds (Bower, Foster, et al., 2020; Schmitt et al., 2018) and thus might go some way to closing attainment gaps when children start school.

#### Extrinsic spatial skills

To date, there are no published studies that have specifically investigated the relationship between extrinsic skills and mathematics in young children. However, it has been shown that spatial scaling training at 8 years leads to improved number line estimation abilities (Gilligan, Hodgkiss, et al., 2019; Gilligan, Thomas, & Farran, 2019), and a number of interventions have trained extrinsic skills alongside intrinsic skills, but again, mainly in samples of older children. For example, Lowrie and colleagues trained secondary school children using lessons that included navigation activities (Lowrie et al., 2017) and in a subsequent study, scaling, route knowledge and perspective taking activities (Lowrie et al., 2019), alongside intrinsic activities such as mental rotation. These studies report improvements in both spatial and mathematics ability. However, because these interventions trained a range of spatial skills beyond extrinsic skills alone, it is not possible to pinpoint the direct impact of the extrinsic components of the training.

Young children can develop their extrinsic spatial skills through activities that require spatial navigation (see Nazareth et al., 2019), using or generating simple maps, imagining different perspectives and scaling between differently sized spaces. For example, small world play can be used to develop a sense of scale and can be used to help children to visualize environments from different viewpoints. Further research is needed to determine how these skills associate with mathematics in early childhood.

#### Spatial language and gesture

Spatial language – words like ‘on’, ‘above’ and ‘next to’ – helps children to: encode and remember information (Feist & Gentner, 2007), draw their attention to relevant spatial dimensions (Bower, Zimmermann, et al., 2020; Farran & O'Leary, 2016), improve their conceptual understanding (Farran & Atkinson, 2016) and highlight the spatial relations that underlay mathematical concepts (Mix & Cheng, 2012). Similarly, gesture, which involves using the hands or arms to enrich communication (e.g., using wide arms to demonstrate ‘big’), can be used to aid children's understanding of spatial words and spatial concepts that may otherwise be difficult to understand. Gesture can also be used to trace the outlines of shapes, thus drawing attention to spatial properties, or to gesture a motion (such as rotating a puzzle piece).

There is evidence that the level of exposure to spatial language in toddlers is associated with their later spatial language and spatial skills at 5 years (Pruden et al., 2011) and that spatial language comprehension at 3 years is predictive of spatial skills at 5 years (Verdine et al., 2017), as well as concurrent mathematics performance (Bower, Foster, et al., 2020; also see Gilligan‐Lee et al., 2021). Although there are few studies that have used gesture with young children, those that have, consistently report positive causal associations. For example, Young et al. (2014) report that jigsaw training with 4‐ to 5‐year‐olds was most effective in improving jigsaw play when the training involved using both gestures and spatial language, compared to training using spatial language alone. Bower, Zimmermann, et al. (2020), demonstrated with 3‐ to 4‐year‐olds that puzzle training with spatial language was more effective in improving spatial ability, shape name knowledge (and broader mathematics ability in low SES children) than puzzle training alone or puzzle training with gesture. Taken together, these two interventions demonstrate the value of using spatial symbols such as language and gesture when implementing spatial activities with children.

### Translating research into practice

Despite what we know about spatial reasoning, it is not clear how successfully this research has transitioned into practice. There are several possible reasons for this. First, research papers are reportedly found to be difficult to access due to overly technical language (Vanderlinde & Braak, 2010). It is also often unclear to practitioners what specific and practical applications the findings could have, making it difficult for them to tease out what the research means for them (Jamaludin et al., 2019). This highlights that the issue of how research findings are translated into educational practice goes beyond how researchers present information in journal articles (Farley‐Ripple et al., 2018) and shows that effort is required to facilitate discourse between researchers and practitioners regarding the design, content and practical applications of research.

Second, limited time and restricted access to research‐based resources is a pertinent issue. Discussion of the issues of chronic workload and limited funding in early childhood education is beyond the scope of this article. Many practitioners report not having enough time to read published papers and reports (Gore & Gitlin, 2004), which they can also find too complex, ambiguous and/or descriptive (Vanderlinde & Braak, 2010). Finally, practitioners are restricted by the curriculum, assessments and regulatory policies at the national and local level. This results in limited freedom with respect to time and cost in implementing suggestions from research (Graves & Moore, 2018). Thus, practical, accessible suggestions that are close‐to‐practice and can be easily implemented into their planning and classroom activities are needed.

Practitioner's beliefs and definitions of constructs, including mathematics and science, are an important factor in translating research into practice because of how they can shape teaching practice. In the context of mathematics, it is suggested that teacher's beliefs can be content‐specific, as well as affective in nature, and these are both thought to influence instructional practice (Schoen & LaVenia, 2019). Teacher's confidence in mathematics practice is also found to be associated with students' level of confidence (Stipek et al., 2001). Moreover, teacher's anxiety about spatial reasoning is negatively associated with students' mental rotation ability (Gunderson et al., 2013). It is, therefore, important to not only translate knowledge of spatial reasoning effectively, but to consider teacher's beliefs and confidence in the concept of spatial reasoning.

### The current study

To provide informative resources for practitioners and effectively translate research findings, there is a need to establish: the current state of practitioner knowledge of spatial reasoning, if and how this knowledge translates into practice, and what the specific barriers and opportunities are for increasing the use of spatial reasoning in early childhood practice. To provide these practitioner insights on spatial reasoning, we conducted a mixed methods study. The first aim was to investigate practitioners' knowledge and implementation of spatial reasoning using a questionnaire study (Study 1). The second aim was to investigate opportunities and barriers to supporting children's spatial reasoning in a qualitative study using focus groups (Study 2). Through collaboration with practitioners, we provide a unique insight into spatial reasoning from inside the home, nursery and/or classroom setting from the perspective of the practitioner.

## STUDY 1: PRACTITIONER'S KNOWLEDGE AND IMPLEMENTATION OF SPATIAL REASONING

### Materials and methods

#### Participants

Participants were recruited using social media, word of mouth and through the networks of the research team. Ethical approval was obtained from the University ethics committee and participants consented to take part using an online consent form. A total of 94 participants completed the entire questionnaire (92 females, number of years working in education: *M* = 16.87, *SD* = 9.41, range = 2–42 years). In this sample, 85 participants (90.40% of sample) reported their ethnic group as White British, other ethnic groups reported were: White Irish, White Other, Mixed Other, Indian, Pakistani, Chinese and Prefer not to say. Most participants reported their main role as either Reception class in primary school (*N* = 31, 33.00% of sample), Key Stage One class in primary school (*N* = 21, 22.33% of sample), Pre‐school, playgroup or not‐for‐profit (*N* = 11, 11.70% of sample) or Nursery class in primary school (*N* = 10, 10.60% of sample). Other main roles reported were Childminder/home‐based practitioner, Private day nursery, Nursery school, Special educational needs setting, Other (e.g., early years advisor).

Participants were split into two practitioner groups, (a) practitioners working with children from birth to 4 years in non‐statutory settings (e.g., nursery, childminders) and (b) practitioners working with children from 4 to 7 years in statutory school‐age settings (e.g., schools). There were 35 participants (34 female) in the birth to 4 years group (number of years in education: *M* = 18.34, *SD* = 9.29, range = 3–40) and 51 participants (49 female, 1 not disclosed) in the 4–7 years group (number of years in education: *M* = 14.78, *SD* = 8.71, range = 2–41). Eight participants did not indicate how old the children were that they worked with, for example early childhood advisor, therefore, they were excluded from any analyses split by practitioner group.

#### Materials and procedure

**FIGURE 1:**
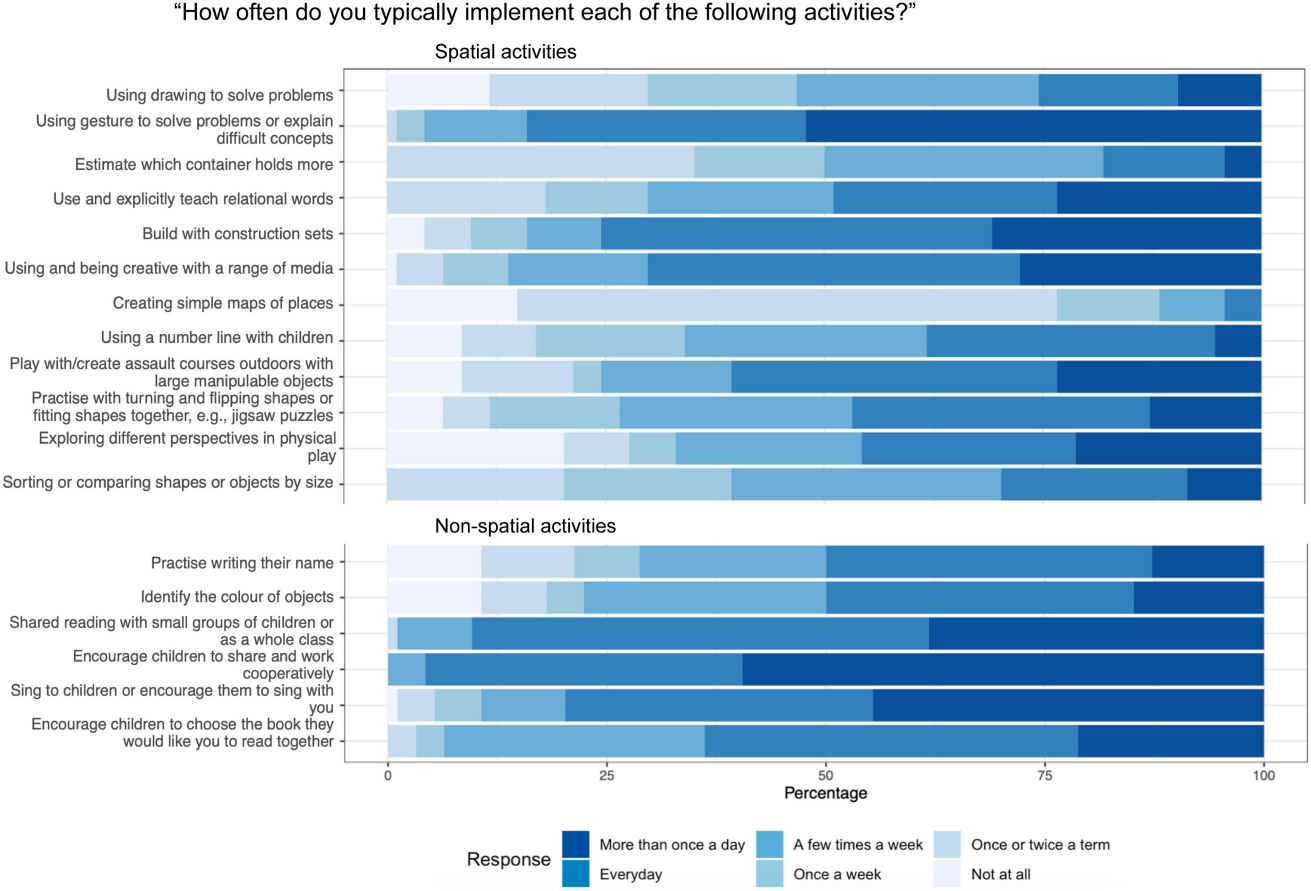
Responses to the question: “How often do you typically implement each of the following activities?” for each of the spatial activities (top) and non‐spatial activities (bottom). The percentage on the *x* axis indicates the percentage of responses per answer: For visualization purposes, we have not included the individual percentages per answer. The stacked bars indicate higher or lower percentages: For example, for the item “Using drawing to solve problems”, the highest percentage of responses was “A few times a week” and the lowest number of responses was “More than once a day”. Note that some items included examples and have been shortened for the purpose of the figure, please see the Supporting Information for full details of the questionnaire items.

**FIGURE 2:**
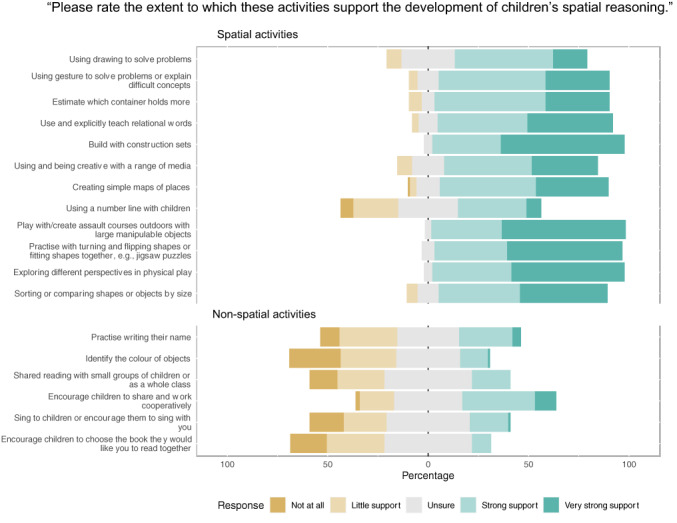
Responses to the statement ‘Please rate the extent to which each of the following activities support spatial reasoning.’ for each of the spatial activities (top) and non‐spatial activities (bottom). The percentage on the *x* axis indicates the percentage of responses per answer: For visualization purposes, we have not included the individual percentages per answer. The stacked bars indicate higher or lower percentages: For example, for the item ‘Using drawing to solve problems’, the response ‘strong support’ has the highest percentage and ‘unsure’ the second highest percentage of responses and there are no responses of ‘Not at all’. Note that some items included examples and have been shortened for the purpose of the figure, please see the Supporting Information for full details of the questionnaire items.

**FIGURE 3:**
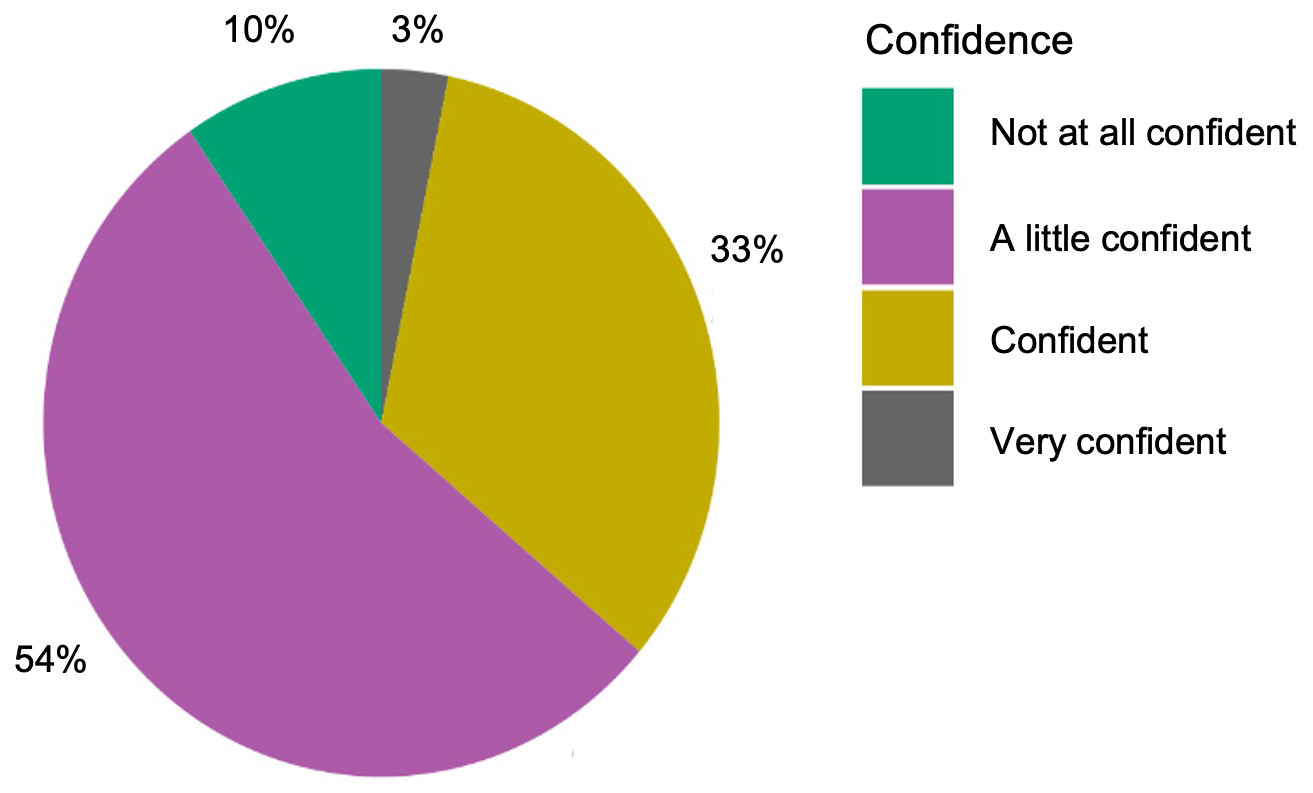
Percentage of responses for each level of confidence in participants' definition of spatial reasoning

A questionnaire was designed to capture practitioners' current knowledge of spatial reasoning and the extent to which practitioners currently implement activities that support spatial reasoning. The questionnaire took approximately 10 minutes to complete and included six questions. The findings of the first three questions are reported here. The remaining questions related to training and resource requirements and will be reported separately. Participants were first asked how frequently they used each of 18 activities (12 spatial and 6 non‐spatial, presented in fixed random order, adapted from Gilligan‐Lee et al. (2022) in their practice using 6 possible response options, ranging from ‘More than once a day’ to ‘Not at all’ as shown in Figure 1. This question was deliberately posed before any questions that asked about spatial reasoning, to avoid introducing bias. Question 2 asked participants how confident they would be in explaining what spatial reasoning is to someone else using four possible response options (see Figure 2) and then to provide a definition of spatial reasoning (open text response). Question 3 asked participants to rate the extent to which the list of activities (as presented in question 1) support the development of children's spatial reasoning. Five response options from ‘Not at all’ to ‘Very strong support for spatial reasoning’ were used, as shown in Figure 3.

#### Data coding and analysis

Quantitative analysis was conducted on the measures derived from questions 1 and 3. For question 1, how often participants are implementing activities, a mean frequency score was derived across all twelve spatial activities and across all six non‐spatial activities. A higher score indicated higher frequency of implementing the activities. For question 3, in which participants rated the extent to which the same activities support spatial reasoning, a mean supporting spatial reasoning score was derived for the 12 spatial activities and for the 6 non‐spatial activities. A higher score indicates stronger perceived support for spatial reasoning. Tests of normality showed variables were normally distributed (*p*s > .05). Previous recommendations when using parametric analyses for data from Likert‐scales are to ensure that there is a minimum of 5 points and to consider non‐parametric if results are close to the significance cut‐off (Carifio & Perla, 2007). The questionnaire items reported here have 6 points and the findings are not close to significance cut‐off, and thus meet these criteria. Nevertheless, non‐parametric alternatives were carried out and we can confirm that the non‐parametric findings did not differ in significance from the parametric analyses reported.

For question 2, we coded practitioner definitions of spatial reasoning qualitatively, according to reference to intrinsic and/or extrinsic skills (framework outlined in Table 1) and whether participants referenced spatial language and/or gesture (see Table 2 for coding framework).

**TABLE 1:** Coding framework for the definition of spatial reasoning

Code	Description
Intrinsic	Manipulation of objects, space, size Manipulatives Visualization/mental imagery/mental images of individual objects and their features Mental transformation Mentally transforming 2D and/or 3D objects
Extrinsic	Exploration/Navigation Moving/manipulating body in space Perspective taking Spatial relations between objects and/or distances Visualization/mental imagery/mental images of how multiple objects interact, or the self‐interacting with objects
Intrinsic and extrinsic	Refers to one or more intrinsic code and one or more extrinsic code
Neither	No reference to either

**TABLE 2:** Additional coding for spatial language and gesture

Code	Description
Spatial language and gesture	Spatial language (e.g., using terms such as ‘under’, ‘above’, ‘big’, ‘small’) Gesture (e.g., to support the use of spatial language such as wide arms indicate ‘big’, hands close together indicate ‘small’; gesturing motor actions such as rotating a jigsaw piece; tracing outlines of shapes)

### Results

#### How often do practitioners implement spatial activities?

A mixed ANOVA with frequency score as the dependent variable, activity (spatial, non‐spatial) as the within‐subject factor and practitioner group (birth‐4 years and 4–7 years) as the between‐subject factor was conducted. This revealed a significant main effect of activity, *F*(1, 84) = 130.39, *p* < .001, $\eta_{p}^{2}$ = .61, due to more frequent use of non‐spatial (*M* = 4.79, *SD* = .67) compared to spatial activities (*M* = 3.97, *SD* = .81). There was also a significant main effect of practitioner group, *F*(1, 84) = 8.65, *p* < .001, $\eta_{p}^{2}$ = .09, which showed practitioners working with children from birth to 4 years (*M =* 4.62, *SD =* .69) implemented both the spatial and non‐spatial activities more often than practitioners working with children from 4 to 7 years of age (*M* = 4.21, *SD* = .90). There was no significant interaction between activity and practitioner group (*F* < 1). Figure 1 displays the distribution of responses per item.

#### How accurately do practitioners recognize activities that support spatial reasoning?

A mixed ANOVA was conducted with the supporting spatial reasoning score as the dependent variable, activity (spatial, non‐spatial) as the within‐subject factor and practitioner group (birth to 4 years, 4–7 years) as the between‐subject factor. There was a significant main effect of activity, *F*(1, 84) = 311.81, *p* < .001, $\eta_{p}^{2}$ = .79 because participants recognized that the spatial activities (*M* = 4.14, *SD* = .46) provide stronger support for spatial reasoning than the non‐spatial activities (*M* = 2.71, *SD* = .75). There was no main effect of practitioner group and no interaction between activity and practitioner group (*F*s < 1), see Figure 2 for distribution of participant responses per item.

#### What is the relationship between the frequency of implementing spatial activities and perceived support for spatial activities?

We were also interested in whether participant's mean spatial activity score for frequency of implementing each activity and mean spatial activity score for rating each activity as supporting spatial reasoning, were correlated. Pearson's correlations revealed a significant correlation overall, *r*(92) = .280, *p* = .006 demonstrating that those practitioners who implemented spatial activities more frequently were also more likely to perceive those activities as supporting spatial reasoning.

#### Defining spatial reasoning

Participants were asked to rate how confident they would be in their definition of spatial reasoning if they were to explain the concept to someone else. Over half reported that they were ‘A little confident’ (54%), around a third reported that they were ‘Confident’ (33%) and 10% were ‘Not confident at all’ with only 3% reporting that they were ‘Very confident’ in their definition (see Figure 3).

Participants were next asked to define spatial reasoning. This demonstrated that 56% of respondents referred to extrinsic skills, for example: ‘How objects relate to each other in space and our perception of ourselves in space and how we relate to objects around us’. By comparison, 21% of participants referred to intrinsic skills in their definitions, for example: ‘Being able to manipulate objects and think about the reason for the manipulation’. Only 10% of participants referred to both extrinsic and intrinsic skills when defining spatial reasoning, for example: ‘How things including ourselves, relate to the physical space around us, the ability to imagine things in 3 dimensions, helps children to visualise things in their heads…’. Finally, 13% of participants either stated they did not know how to define spatial reasoning, or their definitions did not include any reference to extrinsic or intrinsic skills, for example: ‘How we move in our surroundings to achieve what we want, for children it may be touching something, building a tower of bricks etc.’.

### Discussion

When asked which activities supported spatial reasoning, practitioners' scores were higher for most spatial activities compared to non‐spatial activities. Despite this recognition, practitioners implemented spatial activities less frequently than non‐spatial activities. Thus, as a group, whilst practitioners could recognize spatial activities, they did not prioritize implementing these activities. Finally, while most of the sample gave ample definitions of spatial reasoning (only 13% did not provide a clear definition), the majority of participants reported being only ‘a little confident’ in their definition or ‘not at all’ confident in their definition. Given that practitioner confidence is associated with student's level of confidence in mathematics (Stipek et al., 2001), and practitioner anxiety about spatial reasoning is negatively associated with students' mental rotation ability (Gunderson et al., 2013), our findings suggest that action is required. Specifically, that discourse between researchers and practitioners is required to (a) develop knowledge of how spatial reasoning contributes to learning specifically in mathematics and (b) develop close‐to‐practice applications of research.

The spatial reasoning activities we presented included building with construction sets, creating maps and using gesture and spatial language. Construction sets (e.g., Lego, Duplo) involve intrinsic spatial skills and tap into skills such as mental rotation, visuospatial working memory and part/whole understanding, all of which are important for mathematics (McDougal et al., 2022). Creating maps taps into extrinsic spatial skills such as perspective taking and spatial scaling, which has shown some relationship to maths in older children (Gilligan et al., 2019). Interestingly, number lines were not perceived to support spatial reasoning in this group of practitioners. Number lines are a spatial representation of number in which numerical relationships are represented spatially, which requires intrinsic spatial skills (Möhring et al., 2018). Children need to understand this spatial representation to answer, ‘Where does 3 go?’, and use proportional reasoning to do so (Gilligan, Thomas & Farran, 2020). Thus, despite evidence suggesting that number lines support spatial reasoning, better translation of the research findings is required to improve knowledge around how spatial reasoning ability can contribute to number line performance.

Despite the relatively low frequency of *implementing* spatial activities, we found that for spatial activities, higher ‘supports spatial reasoning’ ratings were associated with a higher frequency of implementing those activities. Whilst correlations are not causal this finding could mean two things. It may suggest that participants were more likely to implement spatial activities if they recognized that they support spatial reasoning. It could also mean that practitioners who use more spatial activities in their classroom have a better understanding of the value of these activities for supporting spatial reasoning development. This suggests that if practitioners were provided with the resources and training to increase their knowledge of what spatial reasoning is, its importance for maths and how to support children's development of spatial reasoning, this would assist them in implementing spatial activities more frequently in their practice.

Related to the suggestion above, our data demonstrate that most practitioners were only ‘A little confident’ or ‘Not at all confident’ in their definition of spatial reasoning. When asked to define spatial reasoning, most practitioners only referenced extrinsic skills and 13% of practitioners either did not know how to define spatial reasoning or stated incorrect explanations of spatial reasoning. Mathematics content‐specific practitioner beliefs influence their instructional practice (Schoen & LaVenia, 2019), thus a limited definition of spatial reasoning represents a barrier to the accessibility of research findings to practitioners. In Study 2, we present findings from focus groups in which practitioners discussed their understanding of spatial reasoning, how they support spatial reasoning and the opportunities and barriers to doing so.

## STUDY 2: INVESTIGATING OPPORTUNITIES AND BARRIERS TO SUPPORTING SPATIAL REASONING SPATIAL REASONING

### Method and materials

#### Practitioners

Two focus groups were conducted. These formed part of a Patient and Public Involvement project between researchers, education consultants and practitioners to design an accessible and informative spatial reasoning toolkit. The resultant toolkit can be found at: https://earlymaths.org/spatial‐reasoning/. Practitioners were recruited via social media and contacts of the Early Childhood Mathematics Group. Practitioners completed online consent forms prior to taking part in the focus groups. Five practitioners (3 females) working with children from birth to 4 years participated in the first focus group and four practitioners (4 females) working with children from 4 to 7 years participated in the second focus group. Practitioners in the birth to 4 years group had worked in education for an average of 28 years (*SD* = 18.38, range = 15–41, *N* = 2 respondents to this item only) and practitioners in the 4 to 7 years group worked in education for an average of 13.75 years (*SD* = 14.22, range = 5–35, *N* = 3 respondents to this item only). Most participants reported their ethnic group as White British and a minority as Black or Black British African. The roles of practitioners in the birth to 4 years group included early years teachers, nursery practitioners and nursery school special educational needs coordinators. In the 4–7 years group, practitioner roles included Key Stage One (aged 4–7) teachers, reception teachers and senior leadership team members.

#### Materials and procedure

Participating practitioners attended one online focus group depending on whether they worked with children from aged birth to 4 years or 4–7 years. Each focus group followed the same interview schedule (see Supporting Information) and was led by a researcher. The researcher remained neutral and only intervened to engage members of the group in the discussion or to move the discussion onto the next topic. The focus group began with a 5‐min presentation from the researcher explaining the purpose and the format of the questions. Practitioners were reminded that they were welcome to input as much or as little as they wanted to. At the end of the discussion, the researcher summarized the main points of the discussion and the practitioners had the opportunity to make any final comments. Focus groups were audio and video recorded and transcripts were generated.

#### Focus group analysis approach

The qualitative data from each of the focus groups was analysed separately and is reported as such below; similarities and/or differences between the themes are interpreted in the discussion. Thematic analysis was conducted; this is a data‐driven approach to analysis that emphasizes the researcher's generative role in determining meaningful themes that represent patterns of shared meaning and concepts (Braun & Clarke, 2006, 2019). Therefore, rather than searching for predefined themes, themes were generated based on the practitioners' responses, within the context of our key research questions. These included themes that arose from the group interactions (social constructive approach), as well as personal perspectives arising from individual verbal content (individualistic perspective; Ryan et al., 2014).

Two researchers coded each focus group independently and they both constructed their own themes from the codes. They then came together to discuss, review and determine the final themes. The researchers largely agreed on the themes constructed from the 4 to 7 years focus group; however, there was some disagreement for the birth to 4 years focus group themes. Therefore, a third researcher's analysis of the birth to 4 years focus group data was used to finalize the themes.

### Results

**FIGURE 4:**
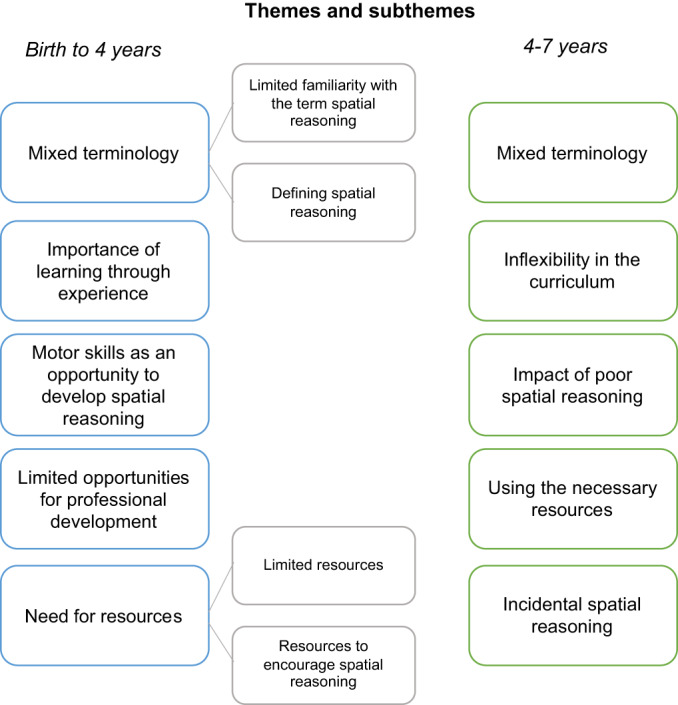
Themes and subthemes generated from the focus groups with the birth to 4 years group (left, blue and grey) and the 4–7 years group (right, green)

There were five key themes identified in the birth to 4 years focus group: Mixed terminology (subthemes: Limited familiarity of the term spatial reasoning and Defining spatial reasoning), Importance of learning through experience, Motor skills as an opportunity to develop spatial reasoning, Limited opportunities for professional development, Need for resources (subthemes: Limited resources and Resources to encourage spatial reasoning). In the 4–7 years group, there were also five key themes identified: Mixed terminology, Inflexibility in the curriculum, Impact of poor spatial reasoning, Using the necessary resources and Incidental spatial reasoning. See Figure 4 for outline of themes and subthemes. In the next sections, we elaborate on each of the themes with supporting quotes and interpretation.

#### Themes: Birth to 4 years focus group

##### Theme: Mixed terminology

###### Subtheme: Limited familiarity of the term spatial reasoning

Practitioners were more familiar with the term spatial awareness than spatial reasoning. If they had come across the term spatial reasoning, this was in the context of mathematics.

I don't think I've ever used the term spatial reasoning… I've used the word spatial awareness a lot… when I've written reports with the children (Practitioner 4)

###### Subtheme: Defining spatial reasoning

The conversation around spatial reasoning in the practitioner setting and what spatial reasoning is, mainly focused on spatial relations between others and objects. Practitioners talked about fitting things together, such as jigsaw pieces or fitting washing on the washing line. Others also discussed how children must consider the space between themselves and other children in everyday tasks, for example when lining up in class or painting together on a large sheet.

… the understanding of the concept of space in relation to objects, but also in relation to ourselves, so if we're thinking about ourselves it's what we can fit into, so suppose if children are building a den and there's four children it's having that understanding of how big that den needs to be (Practitioner 1)

##### Theme: Importance of learning through experience

Throughout the discussion, practitioners referred to learning through experience. This was in the context of creating opportunities for children to learn and practice by fostering independence, such as allowing them to set the table for lunch or engage in risky play like climbing a tree. This was also discussed in the context of how later abilities depend on the earlier development of spatial reasoning, for example it was suggested a child might apply spatial reasoning when learning to write and fitting the words on the line of a page.

I'm thinking of block building and just that moment when children learn or understand how to build a building that doesn't fall down, so they might have a lot of times when you know they're building a tower and keeps falling down, and then they begin to understand if they make the base bigger … I think you know we offer lots of experiences in early years that gives children a chance to try something and it's fine if it doesn't work that's all part of the learning (Practitioner 5)

##### Theme: Motor skills as an opportunity to develop spatial reasoning

When discussing how spatial reasoning might be defined and implemented in practice, many referred to motor skills and body awareness, as well as how physical activity, such as Physical Education (PE) or dancing, can form a way of implementing spatial reasoning in the classroom.

Even just doing things like a very simple sports day in the summer and knowing that you've got to stay in your line and the children find it hard that they've got to stay in their line, and they've got to not cross over with other children and that can be quite a good way to practice [spatial reasoning] (Practitioner 4)

##### Theme: Limited opportunities for professional development

Many practitioners expressed that they themselves were a barrier to implementing spatial reasoning in settings due to limited training and subject knowledge. It was argued that training was very important given that children might have fewer opportunities in spatial reasoning at home, due to busy parents and limited resources and/or interaction with parents.

I know when I initially did my level three then went on and did my foundation degree and teacher training and everything, spatial reasoning wasn't mentioned very much (Practitioner 1)

##### Theme: Need for resources

###### Subtheme: Limited resources

Limitations in the availability of appropriate resources were suggested to negatively impact children's spatial reasoning development. In some cases, this was with respect to the COVID‐19 restrictions in place at the time of the focus groups (July 2021) in that fewer resources can be used because of cleaning requirements and keeping distance. Limited finances for resources, especially outdoor activities, and lack of space at school and in the classroom were also suggested to restrict children's spatial reasoning development.

finances or just a lack of resources can sometimes mean that you don't get a great breadth of experiences … we maybe don't have some of those bigger construction kind of things that you could really build some sort of big models in teams (Practitioner 4)

###### Subtheme: Physical resources and spatial reasoning

Physical resources were also discussed, for example resources for learning shapes and patterns, playing number games and measuring and estimating capacity, as activities that support spatial reasoning. Construction areas and block play were also suggested to encourage children to understand how pieces fit together.

When children are sort of working out number puzzles… that must engage a certain degree of spatial reasoning … we practice that concept by talking to children about what they're doing and can they see the pattern … and getting them to guess what might be next (Practitioner 5)

#### Themes: 4–7 years focus group

##### Theme: Mixed terminology

The familiarity with the term spatial reasoning was mixed; some were more familiar with the concept of spatial awareness, but others were aware of the term spatial reasoning in reference to the new Early Years Foundation Stage framework (Department for Education, 2021). Practitioner's definitions largely referred to awareness of space between objects and others, but also included reference to the ability to visualize and remember spatial information that could later be used in problem solving.

understanding around how we perceive, use and manipulate objects, in a certain area or space. I think that was the best way I could summarize it and by objects that could mean other people, like it could mean yourself, or it could mean another body like yourself within a group or it could mean stacking blocks. (Practitioner 2)

##### Theme: Inflexibility in the curriculum

Many practitioners felt it was important to include spatial reasoning in their teaching practice but there were differences in opinion as to whether time should be carved out to explicitly teach spatial reasoning, or whether teachers should be thinking about how to integrate activities into their current teaching that might aid spatial reasoning development. Issues were raised with how the curriculum can present difficulties in including spatial reasoning in teaching. This included limited flexibility in settings where teaching is prescriptive, limited fluidity in teaching as children get older and, therefore, less time to focus on spatial reasoning skills, expectations from senior leadership to be meeting curriculum goals and limited subject knowledge from both senior leadership and teachers regarding the importance of spatial reasoning.

SLT [Senior Leadership Team] priorities … [spatial reasoning] is not always at the top of the pecking order in terms of what is important right now … I know my current senior leadership team, if I said to them we need to discuss the importance of spatial reasoning, they would probably say no (Practitioner 1)

##### Theme: Impact of poor spatial reasoning

Practitioners discussed how poor spatial awareness, that is when children struggle to judge the space around them, can negatively impact children's behaviour, relationships and learning.

[spatial reasoning] kind of underpins everything else because if [children] are unaware of the space that surrounds them … if they're not in the right sort of zone spatially for learning … if the spatial reasoning is poor then that, ultimately, is going to prohibit them from learning later on down the line so it's kind of like the foundation to learning really. (Practitioner 1)

##### Theme: Using the necessary resources

A variety of resources were identified as being implemented in current practice that might support spatial reasoning, including construction, block play and using manipulatives in mathematics. It was also discussed that there are limits to how these resources might be used; firstly, with respect to time and behaviour management and secondly with respect to the expectation that such activities should be replaced by more formal work, for example writing in books, as children get older.

I was running a maths intervention, at year three level, and even then it was the manipulatives that really got through to these students so it's clear that they really want to use them, and may find them useful and it's just finding the opportunity to teach them how to use these things properly where sometimes it feels like there isn't the space to. (Practitioner 3)

##### Theme: Incidental spatial reasoning

This theme arose as part of the group interaction; throughout the discussion, practitioners began to realize that spatial reasoning is embedded in many of the activities that children do in the classroom, for example lining up for lunch or cutting and sticking work in books.

I think [spatial reasoning] is really, really important, and I think having a discussion like this actually you realize that you are doing it. So much of the time that you know it's not part of the curriculum, it is about lining up and it's all those kind of things, and it's about helping a child actually function, you know, in the world, I mean it's a really important skill to be able to do (Practitioner 5)

### Discussion

The focus group study provided a more in‐depth understanding of practitioners' perspectives on supporting spatial reasoning. Practitioners working with children from birth to 4 years were less familiar with the term spatial reasoning compared to other terms such as spatial awareness, and their descriptions of spatial reasoning were focused on extrinsic skills; fitting objects together and children's awareness of the space between themselves and other children. Interestingly, practitioners working with children from 4 to 7 years also focused on spatial awareness and extrinsic skills, but some also referred to intrinsic skills, such as the ability to visualize and mentally rotate objects to solve problems. Together, this is broadly in line with the Study 1 questionnaire findings in that the majority (66%) of practitioners only referred to extrinsic skills in their definitions. Conversely, the spatial reasoning literature has primarily focused on intrinsic skills. This has demonstrated that the development of intrinsic skills from at least age 3 years is particularly important for later spatial reasoning ability and mathematics (Atit et al., 2021; Hawes et al., 2022; Schmitt et al., 2018), yet there is very little knowledge of the relationship between extrinsic spatial skills and mathematics in young children. This difference in the balance of practitioner definitions versus research focus demonstrates a specific gap between research and practitioner knowledge where better translation of research findings into practice is clearly needed.

Throughout the discussion, practitioners in the birth to 4 years group referred to how important it is to foster independence in young children; allowing them to learn by practicing and making mistakes (theme: Importance of learning through experience). Often their descriptions of activities that support spatial reasoning focused on motor skills, including formal education such as PE and moving around the classroom such as lining up in a straight line (theme: Motor skills as an opportunity to develop spatial reasoning). The emphasis on allowing children to learn and practise is in line with what we know about exploration of space in the early years. That is, it has been shown that the extent to which toddlers explore large‐scale space at 20 months positively contributes to their spatial processing and spatial language abilities one year later (Oudgenoeg‐Paz et al., 2015). Thus, our findings indicate that practitioners working with children from birth to 4 years recognize that there is ample opportunity for activities that support spatial reasoning, which is in line with the current literature.

On the other hand, a prominent focus for practitioners working with children from 4 to 7 years was how spatial reasoning is implemented in practice in relation to the curriculum (theme: Inflexibility in the curriculum). There was discussion as to whether practitioners should be explicitly teaching spatial reasoning skills or whether spatial reasoning activities should be embedded in the teaching of the current curriculum, for example using manipulatives in mathematics. Practitioners expressed that limited subject knowledge of how spatial reasoning supports learning inhibits their ability to implement spatial reasoning in their practice. Our findings show that teachers have a good understanding of the tools that could support spatial reasoning in the curriculum; however, their freedom to be flexible in their teaching practice is restricted by poor translation of current research and pressure to deliver curriculum goals.

The theme of Impact of poor spatial reasoning was unique to the 4–7 years group; this was a theme that emerged throughout the discussion as practitioners started to connect the concept of spatial reasoning with the behaviour and characteristics of the children that they work with. This is also echoed in the theme of Incidental spatial reasoning. As the discussion developed, practitioners noticed all the different ways that spatial reasoning underpins a range of classroom activities, such as lining up for lunch. This demonstrates that, when given the opportunity to reflect, practitioners have a good grasp of how spatial reasoning interacts with children's learning and development.

## GENERAL DISCUSSION

This mixed methods study was conducted to investigate practitioner's perspectives on spatial reasoning in the classroom. The key aims of the study were to establish the current state of practitioners' knowledge of spatial reasoning and what the opportunities and barriers are to supporting spatial reasoning in the classroom. Taken together, studies 1 and 2 demonstrate that practitioners have knowledge of what supports spatial reasoning in the classroom; however, guidance is needed to harness that knowledge and help navigate competing priorities in educational settings to identify opportunities in current practice to support spatial reasoning.

Limited practitioner knowledge was identified in both studies (low confidence and incomplete definitions in Study 1 and the Lack of opportunities for professional development theme in Study 2). For researchers to bridge the research‐practice gap, it is important to ascertain the specific aspects of spatial reasoning that practitioners are trained in. This will identify gaps and requirements for appropriate training resources and guidance on how to develop the professional training that practitioners receive. Despite raising the issue of a lack of professional knowledge of spatial reasoning, practitioners outlined a variety of resources that they use to encourage spatial reasoning. This demonstrates that practitioners are implementing appropriate activities to support spatial reasoning but are not necessarily identifying it as spatial reasoning, perhaps not acting to extend the spatial learning involved in these activities. This is in line with the lack of confidence highlighted in Study 1, which showed that most practitioners reported little to no confidence in their definition of spatial reasoning. As outlined in the introduction, teacher's beliefs and anxiety about mathematics and particularly spatial reasoning can influence not only their instructional practice but also student's confidence and their mathematical ability (Gunderson et al., 2013; Stipek et al., 2001). Therefore, providing practitioners with resources that highlight the opportunities for spatial reasoning development found in the activities they are already doing, would provide a practical guide and increase their confidence in how to support spatial reasoning in their settings. This suggestion tallies with our observation in the 4–7 years focus group where there was evidence that the more practitioners discussed spatial reasoning, the more they began to connect the concept of spatial reasoning with classroom activities and children's behaviours. Considered alongside what we know about how teacher's beliefs influence practice, researchers outlining how current practices might or might not support spatial reasoning abilities could be an avenue for encouraging teacher's confidence in supporting spatial reasoning in their practice.

Resources should focus on how the activities and current resources can be used in a way that encourages spatial reasoning, as research has suggested that the quality of support during activities is more important for spatial performance than the quantity of activities (Casey et al., 2014; also see Jirout & Newcombe, 2015). Alongside this, it was suggested that a lack of finance might have a negative impact on supporting spatial reasoning in young children. This might reflect the allocation of resources to areas perceived to be more central to the curricula and the need for practitioners to work within those specific targets (Graves & Moore, 2018), suggested by comments about the priorities of senior leaders in the respective settings. Whilst the best way to combat this lack of resources is to raise awareness of the importance of spatial reasoning at a policy level, a short‐term response is to draw attention to evidenced‐based activities that do not rely on costly physical resources that can be embedded into current pedagogical practice. Good examples are the use of gesture and spatial language, as well as building with cardboard boxes, creating maps and cutting magazine pictures or greetings cards into jigsaws. Such techniques are simple cost‐effective ways to support children's spatial reasoning.

### Limitations

The present study had several limitations. First, the items included in the questionnaire. Practitioners in the birth to 4 years group implemented more spatial and non‐spatial activities than the practitioners in the 4–7 years group. This suggests that our choice of activities overall was more appropriate for younger children. On the other hand, the items, which showed the lowest implementation frequency overall appear to be items that are less suitable for youngest children, such as making simple maps, perspective taking and practice writing names. Whilst this did not appear to confound our main aim of comparing spatial and non‐spatial items, a more effective set of items would cover the full range of ages from birth to seven years.

The second limitation was the demographics of the sample as most of the practitioners were white and female. Whilst practitioner demographics in England are not available for pre‐school practitioners, government data for School teachers in England suggests that our sample is not representative. In 2019, 85.7% of teachers were White British and 75.8% were women (GOV.UK, 2022). Therefore, further research with a broader sample would increase generalisability. The third limitation is the narrow distribution of roles in the focus groups, particularly in the 4–7 years group as this did not include any junior team members, for example support staff. Exploring the perspectives of practitioners occupying a variety of positions is important as support staff and teachers differ in their communication with children. Specifically, support staff in schools engage in more one‐to‐one interactions, and children are more likely to initiate, respond and sustain interaction with support staff, creating a different dynamic than is present between the children and class teacher (Blatchford et al., 2013). In addition, support staff in schools tend to work more closely with children with special education needs (SEN) than classroom teachers (Blatchford et al., 2013) and so could offer a unique perspective on the barriers and opportunities for developing spatial reasoning within the school‐age SEN population. Therefore, to know where best to direct resources, perspectives from all levels of team members are important.

### Future research

Study 2 formed part of a Patient and Public Involvement project. Informed by these findings among others, a spatial reasoning toolkit was created: https://earlymaths.org/spatial‐reasoning. The next stage for this research will be to trial the spatial reasoning toolkit to determine whether our aim that the spatial reasoning toolkit will improve practitioners' knowledge of spatial reasoning and their ability to effectively support its development, is supported. Specifically, future research should focus on exploring practitioners' impressions of the spatial reasoning toolkit, the perceived utility of the toolkit (i.e., who it benefits and in what capacity), and whether practitioners feel the toolkit will impact their individual practice. Future research could also centre on the implementation of spatial reasoning more broadly across the curricula, as outlined in Gripton (2022).

## CONCLUSION

The current studies suggests that whilst practitioners engage in a variety of activities that support spatial reasoning, the specific spatial and mathematical benefits of these are not always immediately apparent to them. The implementation of these activities in a way that will provide the most benefit to spatial reasoning development, is also constrained by a lack of confidence in their knowledge of spatial reasoning, and a lack of flexibility to implement spatial reasoning, as they conceptualize it. This is at least partly due to limited discourse between researchers and practitioners. Researchers should aim to go beyond providing accessible information and to work alongside education practitioners to better understand their needs in this diverse sector, use this knowledge to derive research questions and ultimately develop close‐to‐practice, evidence‐based resources. To address the gaps highlighted here, informative and accessible resources are needed to broaden understanding of the definition of spatial reasoning and outline opportunities in current teaching practice and available resources to support spatial reasoning.

## AUTHOR CONTRIBUTIONS

**Kathryn E. Bates:** Conceptualization; data curation; formal analysis; investigation; methodology; visualization; writing – original draft; writing – review and editing. **Ashley Y. Williams:** Writing – original draft. **Katie A. Gilligan‐Lee:** Conceptualization; methodology; writing – review and editing. **Catherine Gripton:** Conceptualization; methodology; supervision; writing – review and editing. **Andrea Lancaster:** Conceptualization; methodology; writing – review and editing. **Helen J. Williams:** Conceptualization; methodology; writing – review and editing. **Alison Borthwick:** Conceptualization; methodology; writing – review and editing. **Sue Gifford:** Conceptualization; methodology; writing – review and editing. **Emily K. Farran:** Conceptualization; funding acquisition; methodology; project administration; supervision; writing – original draft; writing – review and editing.

## CONFLICTS OF INTEREST

The authors declare no conflict of interest.

## Supporting information


Data S1.

## Data Availability

Research data are not shared.
