# Supplementary material for: Practitioners' perspectives on spatial reasoning in educational practice from birth to 7 years
Source: Br J Educ Psychol. 2023 Feb 20;93(2):571–90. doi: 10.1111/bjep.12579 (PMC10952197; doi:10.1111/bjep.12579)
Supplement: Supplementary file 1 — Data S1. [file BJEP-93-571-s001.docx]

**Supplementary Materials: Practioner’s perspectives on spatial reasoning in educational practice from birth to 7 years**

**Study 1 Questionnaire**

***Information sheet and consent form removed for the purpose of blinding for review***

**Study 2 Focus group interview schedule**

**The key questions for this study are:**

- What is each participant’s definition / understanding of spatial reasoning?

- Is there variation in participant’s definition / understanding of spatial reasoning?

- How do participants currently implement spatial reasoning activities in their practice?

- What are the barriers to implementing spatial reasoning activities in practice?

- What are the promising opportunities for spatialising activities?

**Introduction for participants**

**-** Welcome

**-** Introduction of the discussant

**-** Why this research?

**-** What will the focus group interview look like? What can you expect?

Invite all to share ideas, opinions and personal experiences.

**-** The role of the discussant. The participants will discuss as much as possible

themselves. The discussion leader is there only to keep up the pace and occasionally

to guide the discussion. There are no right or wrong answers. What matters is what

you think and why you have these perceptions and thoughts. The discussant will be

neutral and objective.

**-** Based on the group discussions, we will summarise our findings.

**-** Do you have any questions before we start?

**Introduction of the theme by discussant:**

Open round to get to know each other and feel comfortable with each other to have an open discussion.

First, we invite participants to express their understanding of spatial reasoning. We ask participants to write down their definition of spatial reasoning as if they had been asked to explain the concept to a friend.

**The group discussions**

Exploration of the definition of spatial reasoning and their understanding of the importance of spatial reasoning in their practice.

The bullet points are used as the main focus of the subsequent discussion (see indicative questions below). Questions cover: Exploration of implementation of spatial reasoning in their practice (exploration of the main bullet points that come up), barriers to doing so, opportunities to spatialise the curriculum, and, where relevant, uncover implementation of spatial reasoning activities in their practice which they had not realised was spatial reasoning.

**Introductory question:**

1. Have you heard the term spatial reasoning before and how frequently?

**Transition question:**

1. Explain in which contexts you might have come across this term or what variations you might have heard (for example, spatial awareness)

**Key questions:**

1. How would you define spatial reasoning?
2. What role do you think spatial reasoning has in classroom learning?

**Transition question:**

1. Where are the current opportunities to include spatial reasoning in the curriculum?

**Key questions:**

1. What are the current barriers to implementing spatial reasoning in the classroom?
2. How important is it to implement spatial activities in the classroom?
3. What types of activities are you currently using?

**[Researcher summarises discussion]**

1. Have I included everything in the summary? Do you want to add anything?

**Instruction for discussant to give a short summary and conclusion of the focus group**

Draw a brief summary of the discussions.

Ask to participants if we forgot to discuss important themes

Any other suggestions?

**Instructions for discussant to stimulate fair discussion:**

Are the outlined perceptions and opinions, shared by the rest of the group?

If no, what are the differences?

Enable quieter participants to express their thoughts and ensure that active participants create space for others.
